# Temporal Interactions between Maintenance of Cerebral Cortex Thickness and Physical Activity from an Individual Person Micro-Longitudinal Perspective and Implications for Precision Medicine

**DOI:** 10.3390/jpm14020127

**Published:** 2024-01-23

**Authors:** John Wall, Hong Xie, Xin Wang

**Affiliations:** 1 Department of Neurosciences, University of Toledo College of Medicine & Life Sciences, Toledo, OH 43614, USA; hong.xie@utoledo.edu (H.X.); xin.wang2@utoledo.edu (X.W.); 2 Department of Psychiatry, University of Toledo College of Medicine & Life Sciences, Toledo, OH 43614, USA; 3 Department of Radiology, University of Toledo College of Medicine & Life Sciences, Toledo, OH 43614, USA

**Keywords:** accelerometer, cortical thickness, human individuality, idiographic, intraindividual, physical activity, precision/personalized medicine, steps/day exercise

## Abstract

Maintenance of brain structure is essential for neurocognitive health. Precision medicine has interests in understanding how maintenance of an individual person’s brain, including cerebral cortical structure, interacts with lifestyle factors like physical activity. Cortical structure, including cortical thickness, has recognized relationships with physical activity, but concepts of these relationships come from group, not individual, focused findings. Whether or how group-focused concepts apply to an individual person is fundamental to precision medicine interests but remains unclear. This issue was studied in a healthy man using concurrent micro-longitudinal tracking of magnetic resonance imaging-defined cortical thickness and accelerometer-defined steps/day over six months. These data permitted detailed examination of temporal relationships between thickness maintenance and physical activity at an individual level. Regression analyses revealed graded significant and trend-level temporal interactions between preceding activity vs. subsequent thickness maintenance and between preceding thickness maintenance vs. subsequent activity. Interactions were bidirectional, delayed/prolonged over days/weeks, positive, bilateral, directionally asymmetric, and limited in strength. These novel individual-focused findings in some ways are predicted, but in other ways remain unaddressed or undetected, by group-focused work. We suggest that individual-focused concepts of temporal interactions between maintenance of cortical structure and activity can provide needed new insight for personalized tailoring of physical activity, cortical, and neurocognitive health.

## 1. Introduction

Adult cerebral cortical structure requires constant maintenance. Distinguished from normal aging of cortical structure over a lifespan of years/decades, normal maintenance, which has received little attention, ensures continuity in cortical structural viability and integrity over short intervals of, e.g., days/weeks.

Working from an idiographic perspective that recognizes the importance of human individuality, precision medicine approaches can potentially impact maintenance of an individual person’s cortical structure and related neurocognitive health through personalized tailoring of modifiable lifestyle factors, including physical activity [1,2,3]. Physical activity has recognized relationships with cortical structure, including thickness of the cortex [4,5,6,7]. Concepts that characterize these relationships come from group-based (nomothetic) approaches. Whether or how current group-based concepts apply to an individual person is basic to precision medicine interests but has not been studied.

We have reported magnetic resonance imaging (MRI) data that micro-longitudinally tracked normal maintenance of cortical thickness in a healthy man at weekly intervals over several months [8,9]. The results suggested that hemispheric mean thicknesses of his left and right cortices underwent reversing incremental and decremental fluctuations that appeared to reflect normal maintenance remodeling/turnover of cortical substrates. These findings raise the possibility that fluctuations in the maintenance of his cortical thickness may interact with ongoing fluctuations in his physical activity.

The present investigation explored this possibility with the use of this individual’s micro-longitudinal cortical thickness maintenance data and concurrent micro-longitudinally tracked physical activity in terms of steps/day. A hope was that an individual person micro-longitudinal approach might complement existing group-based work on relationships between cortical thickness and physical activity in the following ways.

First, existing group work is based on designs that used limited thickness sampling per individual and group analyses of inter-individual average measures of thickness. In contrast, the present study used micro-longitudinal thickness sampling at regular short intervals over several months from a single individual, and intra-individual analyses of thickness. Although unconventional and underused, recent reviews suggest individual-focused analyses are well suited for investigating concepts of human brain–behavior relationships [10,11,12].

Second, group work has focused on the effects of physical activity on cortical thickness, whereas potential reverse direction influences of cortical thickness on physical activity have received little attention. The present micro-longitudinal tracking independently assessed relationships in each direction.

Finally, group work does not systematically address time scales over which activity and thickness maintenance may be related. The present micro-longitudinal analyses were designed to empirically test windows of time when activity and maintenance of thickness were and were not related.

Our study addressed two specific questions that have not been studied at an individual person level. Question 1: Was preceding physical activity related to subsequent maintenance of cortical thickness and, if so, over what times? Question 2: Was preceding maintenance of cortical thickness related to subsequent physical activity and, if so, over what times?

Thickness was used as an index of cortical structural maintenance because it can be objectively and repeatedly measured in an individual person with automated programs. Hemispheric mean thickness was used as a measure because it is appropriate for assessing the spatially ubiquitous nature of the maintenance of cortical structure across the hemisphere. Micro-longitudinal sampling of thickness was used to continuously track maintenance fluctuations at short intervals over months.

Step count was used as an index of physical activity because it: (a) has been used in group-based work on relationships with cortical thickness, (b) is a fundamental unit of energy expenditure in an individual, and (c) has been successfully used as a motivator to increase activity and, thus, is of interest for precision medicine intervention. Accelerometer measures were used because they provide accurate measures of steps. Micro-longitudinal sampling was used to continuously track activity fluctuations at repeated regular short intervals over months.

The present study explores whether maintenance of the cerebral cortical structure of an individual person’s brain interacts with their physical activity. The individual-based focus and questions we address have received little attention; however, we felt an exploratory investigation was justified given (a) their basic significance for understanding temporal interactions between physical activity and maintenance of cortical structure at an individual level and (b) the rare availability of concurrent micro-longitudinal thickness maintenance and physical activity measures over a several-month period in an individual person. 

## 2. Materials and Methods

Details of the studied individual’s medical history and health monitoring measures during the study have been published [8,9,13]. Salient points are briefly re-reviewed next.

### 2.1. Studied Individual

The subject is a 66-year-old man. He was selected based on simple criteria that he had a lifelong good medical history with no chronic illness and was highly motivated to undergo necessary weekly MRI scanning, daily health monitoring, and daily tracking of accelerometer-measured step counts.

### 2.2. Medical History

He has been active across life and had no history of medical problems, childhood abuse, psychiatric illness, concussion, or head trauma. He has been a vegetarian since 2000 and had not used tobacco since 1980 or alcohol since 2000. Prior to those times, he was a sporadic pipe smoker and minimal alcohol consumer. He had never used recreational drugs. Other than wisdom teeth removal and stitches for a minor childhood finger incision, he had no surgeries. The MRI scans indicated no brain abnormalities.

### 2.3. Health during the Study

During the study he experienced no illnesses or trauma, and his daily schedule involved the usual work and home routines. Health monitoring included daily measures of pulse, blood pressure, blood glucose, oral temperature, and weight. Further measures taken at the end of the study included waist circumference, body mass index, hemoglobin A_1c_ (HbA_1c_), C-reactive protein, cholesterol, high-density lipoprotein (HDL), low-density lipoprotein (LDL), and triglycerides. Three physicians independently reviewed these measures and rated all to be within, or approximate, healthy ranges (marginally low pulse (57 ± 3 bpm) and marginally high systolic pressure (124 ± 7 mmHg) on arising in mornings).

Daily questionnaire scores indicated he was within normal ranges for anxiety, depression, and happiness. He did not suffer from sleep disorders, and sleep durations across the study fluctuated within normal adult guidelines. Questionnaire responses indicated he felt mentally alert, healthy, and regularly woke up feeling rested and with high energy. Further assessments indicated he did not suffer from metabolic syndrome and had low allostatic (stress) load. Doppler ultrasound measures at the end of the study indicated that blood flow velocities for cortical arteries were within normal ranges.

Finally, his left and right hemisphere thickness means and ranges were encompassed within thickness measures reported in 11 recent investigations that defined normal adult hemispheric thicknesses with the Freesurfer procedures used in the present study [13].

### 2.4. Prospective Micro-Longitudinal Design

Details of the micro-longitudinal time series measures of hemispheric thickness have been described [8,9]. In brief, MRI scans were made on 22 dates across a 25-week scan period. Except for missed scans at weeks 2, 6, and 7, scans were taken at 1-week intervals on Sundays around the same mid-day start time (mean ± SD: 13:55 ± 2.1 h). On each date, two scans were taken in one session, with removal from the scanner between the first (scan A) and second (scan B) scans (≈5 min interscan time; 11.2 min/scan). This provided 44 hemispheric thickness maintenance measures taken over a total scan time of 8.2 h.

Micro-longitudinal tracking of steps/day was done with a Fitbit One triaxial accelerometer. Activity during windows of time that both preceded and followed scan dates were of interest. Steps/day measures were not available for weeks that immediately preceded, and the first 6 days of, the 25-week scan period. Steps/day activity measures were available each day beginning on day 7 and extending over the remaining days of the scan period, as well as over a further 78 days after the scan period that became of interest. This provided a total of 247 sequential measures of steps/day activity.

### 2.5. MRI Scans, Scan Processing, and Thickness Maintenance Measures

Procedures for MRI scanning, scan processing, thickness maintenance measurement, and measurement error control and assessment have been described in detail [8,9]. Salient points are as follows. T1-weighted scans of the entire brain were made with a 3T GE Signa scanner (164 continuous axial slices, voxel size 1 mm × 1 mm × 1 mm). All scans were made with the same scanner, head coil, and scan parameters. Regular quality assurance tests during the study identified no problems, and scanner upgrades were not done. Scan checks ruled out motion and other artifacts. Scan processing was done with automated FreeSurfer procedures (http://surfer.nmr.mgh.harvard.edu; accessed on 16 January 2023). To treat data from all scans as equal and independent measures, each scan was processed individually without cross-scan registration or averaging. Thickness measures were taken in native space without transformation to a template. Cortical thickness was defined at ≈150,000 vertex locations/hemisphere, and mean hemispheric cortical thickness (mm) was determined for each hemisphere using all vertex measures. To ensure uniform processing, all scans were processed at one time, after collection of all data, using one workstation, operating system, and FreeSurfer (version 4.5.1) program. Cortical borders were visually checked and judged to not require manual correction.

### 2.6. Physical Activity Measures

Step counts were done with one Fitbit One accelerometer worn on the right front waistline between arising in the morning and retiring in the evening, with exceptions for showering. The Fitbit One has been shown to provide valid and reliable counts of step activity [14,15,16]. The individual had no gait irregularities or physical limitations. Steps were free-living and, accordingly, speed, bout durations, and other factors varied with daily needs. Step counts from the device readout were entered into a database each evening and subsequently not accessed or processed until after study completion.

Accuracies of the Fitbit One step counts in our specific subject were of interest. Tests from the studied individual during the study indicated an average 1% (±0.7) difference (836 comparison trials for 28 days) between Fitbit counts and corresponding direct counts of steps taken at variable rates over flat, hilly, irregular, and stair environments. This difference is within reported Fitbit One step count accuracy measures for nonimpaired subjects [14,16,17,18,19,20], including for free-living conditions [21,22,23] and older adult age groups [15,24,25].

Taken together, the above considerations suggest accuracies of daily step counts in the studied individual were high. A limitation is that these step counts come from one individual and, thus, do not represent population measures.

A runs test assessed potential shifting in activity over the study. In addition, Kolmogorov–Smirnov (K-S) uniform distribution analyses tested if step activity differed across days of the week.

### 2.7. Regression Analyses

Regression analyses were used to assess relationships, first, between preceding physical activity and subsequent cortical thickness maintenance (Question 1) and, second, in the reverse direction, between preceding cortical thickness maintenance and subsequent physical activity (Question 2). For both questions, initial analyses pooled left and right thickness measures. Follow-up analyses considered left and right cortices separately. Data were plotted as scatterplots with associated linear regression lines, R^2^, and *p* values. The analyses provided tests of “if-then”, i.e., “if” this occurred earlier, “then” this occurred later, relationships in each direction.

### 2.8. Temporal Analyses

To our knowledge, no prior attempts have been made to examine relationships between maintenance of cortical thickness and steps/day activity over continuous short intervals for an extended period in an individual person. A main interest was assessment of windows of time that did and did not contribute to relationships in each direction. This necessitated trial-and-error empirical testing of variable time windows.

As an a priori plan for systematic testing, a strategy was applied that first tested longer time windows of multiple weeks, which was followed by tests for shorter windows of individual weeks, week segments, and days. This provided tests of relationships over a range of time scales and bracketing of periods that had graded degrees of relationships. This plan was used to successfully assess bidirectional temporal relationships between cortical thickness maintenance and sleep duration in this individual [13].

### 2.9. Significance Levels

Plots of data and regression analyses were done using SPSS. Analyses of different time windows involved different numbers of tests. For analyses involving multiple tests, conservative Bonferroni multiple test-adjusted *p* values were applied, whereby *p* = 0.05/*n*, where *n* = number of tests done in that analysis. Use of Bonferroni adjustments impacted outcomes by reducing chances of false positive results and by increasing the difficulty of obtaining relationships that were interpreted to be significant. Following Bonferroni correction, a test result was considered significant if *p* ≤ 0.05/*n*, and a trend if *p* > 0.05/*n* but ≤0.05. The number of tests associated with Bonferroni correction and the applicable *p* value are indicated in pertinent Results sections.

Given the conservative nature of Bonferroni-adjusted significance levels, trend level outcomes were considered. This was done in view of the possibility that time windows with conservative significant relationships may extend in graded fashion to times with further meaningful but weaker trend-level relationships, and to yet further times with no relationships. This allowed considerations which, on the one hand, did not increase or exaggerate significant outcomes and, on the other hand, did not disregard outcomes at trend levels which may be meaningful for assessing potential graded temporal relationships.

### 2.10. Blind Controls

Controls were applied to support blinded assessments. (1) Thickness measures were not defined until after the study. (2) Similarly, steps/day activity measures were not accessed until after the study. (3) Thickness and activity measures were each processed and defined independently by different investigators who did not have knowledge of the other measure, and were not altered in subsequent analyses.

## 3. Results

### 3.1. Cortical Thickness Maintenance

**Figure 1:**
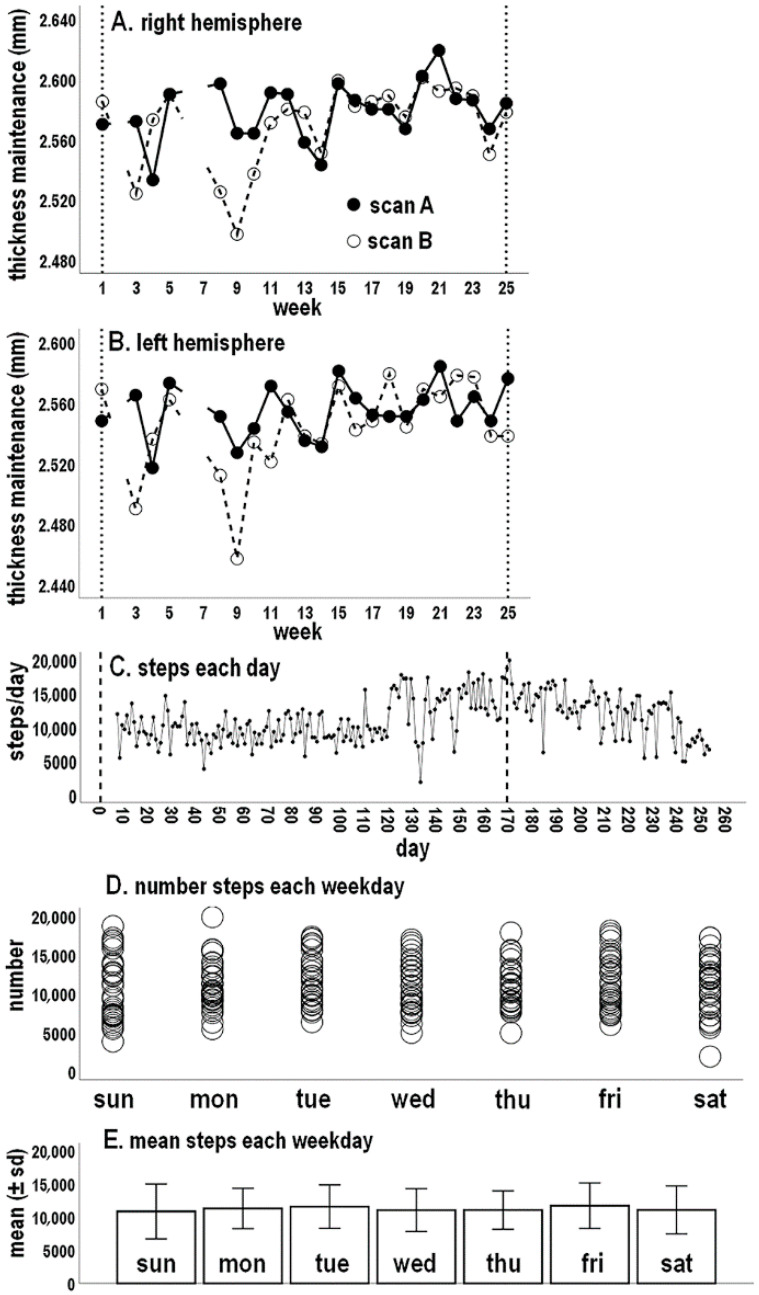
Concurrent micro-longitudinal tracking of maintenance of right (**A**) and left (**B**) hemisphere mean cortical thicknesses and step-per-day activity (**C**). In (**A**,**B**) filled circles indicate scan A, and open circles indicate scan B; breaks in the lines indicate missing data at weeks 2, 6, and 7. Vertical dashed lines in (**A**–**C**) correspond to first and last scan days. Scatterplots of number of steps (**D**) and graphs of mean (±SD) steps (**E**) for each day of the week.

Thickness maintenance profiles for each scan series have been previously described and are plotted across weeks for each hemisphere (Figure 1A,B) [8,9]. Mean thickness measures for each hemisphere, taken on Sundays, underwent reversing incremental and decremental fluctuations from week to week that were not attributable to measurement error. Right thickness was consistently maintained at a higher level on each scan date, leading to a larger mean (±SD) right (2.574 ± 0.025 mm) than left thickness (2.549 ± 0.025 mm). Previous runs tests indicated cortical thickness maintenance of each hemisphere did not progressively shift over time.

### 3.2. Activity across the Study Period

Except for the initial 6 days during which activity measures were not recorded, the number of steps taken each day between the first and last scan dates are shown in Figure 1C. Mean (±SD) activity over this time was 10,714 (±3260) steps/day. In addition, in addressing Question 2, activity for 78 days after the last scan became of interest when assessing temporal relationships between preceding thickness maintenance and subsequent activity. These days became part of the study period (Figure 1C). With these days, mean activity was 11,159 (±3354) steps/day.

A runs test of steps/day over the study period was significant (*p* < 0.001), indicating activity underwent progressive shifting. This appeared largely due to a spontaneous upward shift beginning around day 120, which subsequently drifted down to earlier levels (Figure 1C).

### 3.3. Activity for Different Days of the Week

Scatterplots of the number of steps and graphs indicating mean (±SD) steps are shown for each day of the week (Figure 1D,E). Mean step levels across the 7 days did not differ from a null horizontal distribution indicating a no cross-day difference (K-S, *p* = 0.502). Similarly, standard deviation levels across days did not differ from a null horizontal distribution (K-S, *p* = 0.682). This suggests steps activity was similar for different days of the week.

### 3.4. Question 1: Was Preceding Physical Activity Related to Subsequent Maintenance of Cortical Thickness and, if so, over What Times?

Using the empirical broad-to-narrow time window approach discussed in the Methods, initial analyses focused on activity over preceding broad multi-week periods.

#### 3.4.1. Analysis 1: Activity during Preceding Three-Week Periods vs. Subsequent Thickness Maintenance (*n* = 2 Tests; Bonferroni Adjusted *p* ≤ 0.025) 

**Figure 2:**
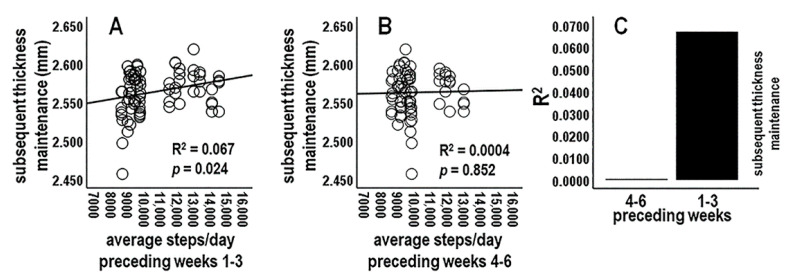
Scatterplots, linear regression lines, and associated R^2^ for relationships between average steps/day during preceding weeks 1–3 (**A**) and 4–6 (**B**) vs. subsequent thickness maintenance. (**C**) R^2^ profile summary for these preceding multi-week periods. The R^2^ profile suggests associations were delayed/prolonged over weeks, with a temporal gradient involving a significant positive relationship for weeks 1–3 and no relationship for weeks 4–6.

There was a significant positive relationship between average steps/day across the immediately preceding 3-week period, i.e., across the three Sunday–Saturday periods for the preceding weeks 1–3, and thickness maintenances on the subsequent Sunday (Figure 2A,C). In contrast, average steps/day across the next earlier 3-week period, i.e., preceding weeks 4–6, was not related to subsequent thickness maintenances (Figure 2B,C).

#### 3.4.2. Analysis 2: Activity during Preceding Individual Week Periods vs. Subsequent Thickness Maintenance (*n* = 4 Tests; Bonferroni Adjusted *p* ≤ 0.0125)

**Figure 3:**
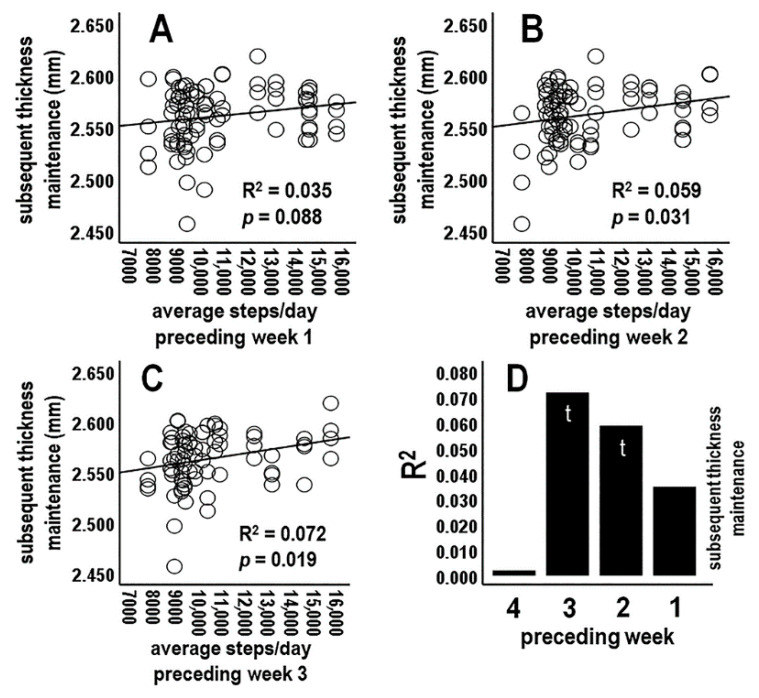
Scatterplots, linear regression lines, and associated R^2^ for relationships between average steps/day during preceding individual week 1 (**A**), 2 (**B**), and 3 (**C**) vs. subsequent thickness maintenance. (**D**) R^2^ profile summary for preceding individual weeks 1–4.

Analyses next focused on average activity for preceding individual week (Sunday–Saturday) periods. There was a nonsignificant relationship between average steps/day for the preceding 1st week vs. subsequent thickness maintenance (Figure 3A,D). Steps/day averages for the preceding 2nd and 3rd weeks each had positive relationships at trend levels (Figure 3B–D). In contrast, the relationship for the preceding week 4 dropped off sharply and was not significant (R^2^ = 0.002, *p* = 0.677; Figure 3D). These results provided a further suggestion of positive relationships between activity at some time(s) during preceding weeks and subsequent thickness maintenance (Figure 3D).

#### 3.4.3. Analysis 3: Activity during Preceding Week Segment Periods vs. Subsequent Thickness Maintenance (*n* = 6 Tests; Bonferroni Adjusted *p* ≤ 0.008) 

The focus next shifted to average daily activity across early week, i.e., Sunday–Wednesday, and latter week, i.e., Thursday–Saturday, segments of preceding weeks 1, 2, and 3.

**Figure 4:**
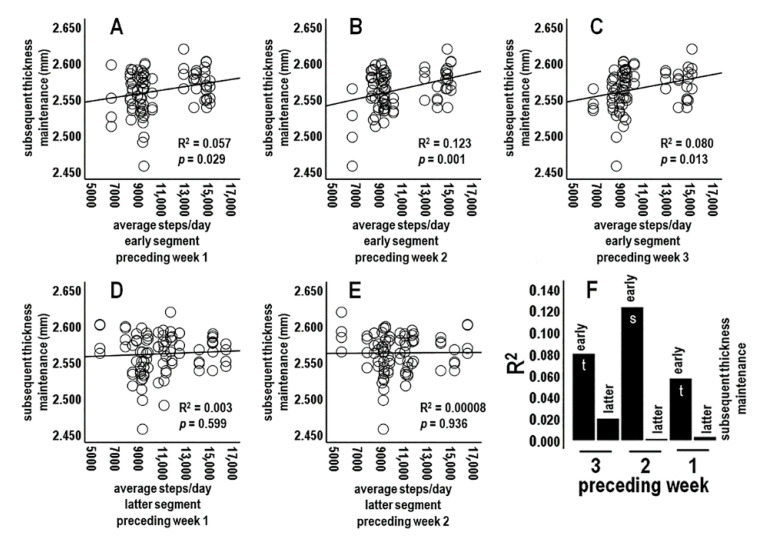
Scatterplots, linear regression lines, and associated R^2^ for relationships between average steps/day during preceding early week segments of weeks 1 (**A**), 2 (**B**), and 3 (**C**), and for preceding latter week segments of weeks 1 (**D**) and 2 (**E**) vs. subsequent thickness maintenance. (**F**) R^2^ profile summary for week segments of preceding weeks 1, 2, and 3. The R^2^ profile suggests a delayed/prolonged gradient in associations involving: first, early week segments that had a significant (s) positive relationship during preceding week 2 and trend (t)-level positive relationships during preceding weeks 1 and 3, and second, no relationships for latter week segments.

Steps/day averages for early week segments were positively related to subsequent thickness maintenances at a significant level for the preceding week 2 and at trend levels for both week 1 and week 3 (Figure 4A–C,F). Noticeably different, activities for latter-week segments (Figure 4D,E) were not related to subsequent thickness maintenance: i.e., compare distributions and regression lines in Figure 4A–C vs. Figure 4D,E, and R^2^ in Figure 4F.

Early week segment positive relationships appeared to be graded, with a relatively higher relationship during the 2nd as compared to the 1st and 3rd preceding weeks (Figure 4F). These positive relationships involved delayed and/or prolonged associations because the early-week activity for preceding week 1 was separated from subsequent thickness maintenance measures by intervening week 1 latter week days, and early week activities for preceding weeks 2 and 3 were separated from subsequent thickness maintenance measures by the intervening weeks (Figure 4F).

#### 3.4.4. Analysis 4: Activity during Preceding Individual Days vs. Subsequent Thickness Maintenance (*n* = 21 Tests; Bonferroni Adjusted *p* ≤ 0.002)

**Figure 5:**
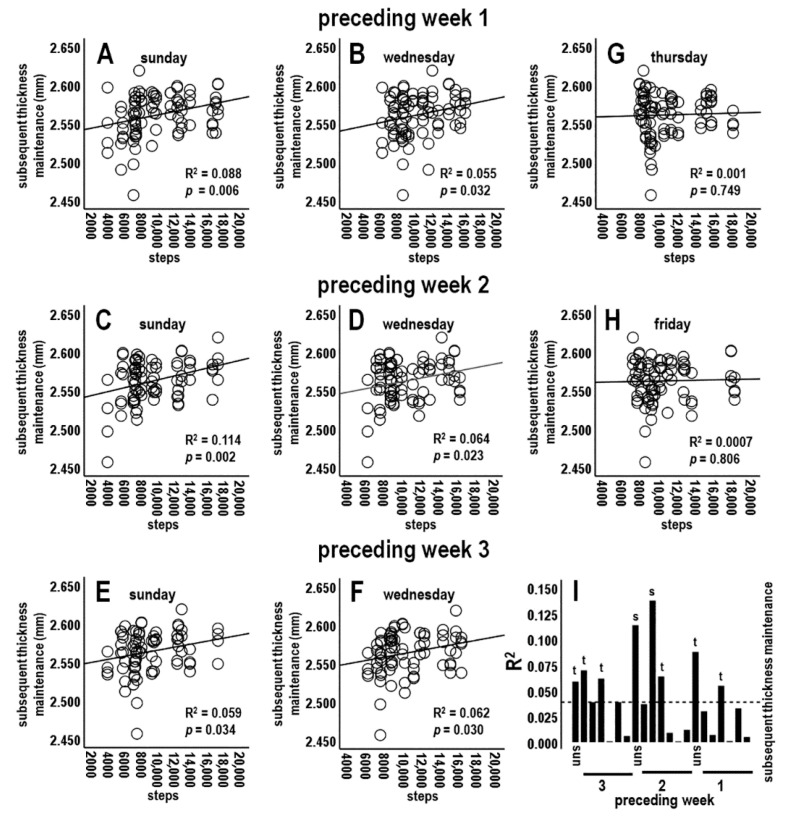
Scatterplots, linear regression lines, and associated R^2^ for relationships between steps during indicated individual early week segment days (**A**–**F**) and latter week segment days (**G**,**H**) of preceding week 1 (top), 2 (middle), and 3 (lower) vs. subsequent thickness maintenance. (**I**) R^2^ profile summary indicating significant (s) and trend (t)-level positive relationships for preceding individual days. R^2^ at and below the dashed line are not significant. In (**I**), for each week Sunday is labeled and followed in sequence by Monday–Saturday.

Steps during individual days of preceding weeks 1, 2, and 3 were next assessed. For preceding week 1, steps on early week segment Wednesdays and Sundays, i.e., the respective 4th and 7th days prior to thickness maintenance measures, were each positively related at trend levels to thickness maintenance on the subsequent Sunday (Table 1; Figure 5A,B,I). Relationships for early week 1 Mondays and Tuesdays were not significant (Table 1; Figure 5I).

For preceding week 2, steps on early week segment Sundays and Tuesdays were each significantly positively related to thickness maintenance on the subsequent Sunday (Table 1; Figure 5C,I). Steps on Wednesdays were positively related at a trend level, whereas relationships for Mondays were not significant (Table 1; Figure 5D,I).

For preceding week 3, steps on early week segment Sundays, Mondays, and Wednesdays were each positively related at trend levels to subsequent thickness maintenance (Table 1; Figure 5E,F,I), whereas relationships for early week Tuesdays were not significant (Table 1; Figure 5I).

In contrast to the above findings for early week segment days, steps on latter week segment Thursdays, Fridays, and Saturdays of preceding weeks 1, 2, and 3 were not related to subsequent thickness maintenance (Table 1; Figure 5G–I). These nonsignificant relationships noticeably differed from positive relationships of early week segment days (e.g., compare positive scatterplot distributions and regression lines for early week segment days in Figure 5A–F to flat distributions and regression lines for latter week segment days in Figure 5G,H).

Analysis 4 results suggest that activity for some preceding early week segment individual days had positive relationships with subsequent thickness maintenance and that these associations were temporally delayed and/or prolonged across shortest to longer times of respectively 4–7 days to 2–3 weeks (Figure 5I). Relationships across days were graded, e.g., with significant to trend-level positive relationships for early week segment days but no significant relationships for latter week segment days (Table 1; Figure 5I). 

#### 3.4.5. Analysis 5: Left and Right Cortex Laterality Analyses (*n* = 42 Tests; Bonferroni Adjusted *p* ≤ 0.001)

The above analyses used pooled thickness maintenances from both hemispheres. To test if preceding individual day activities over preceding weeks 1–3 were related to subsequent thickness maintenances of each cortex, thickness maintenances of right and left cortices were considered separately.

**Figure 6:**
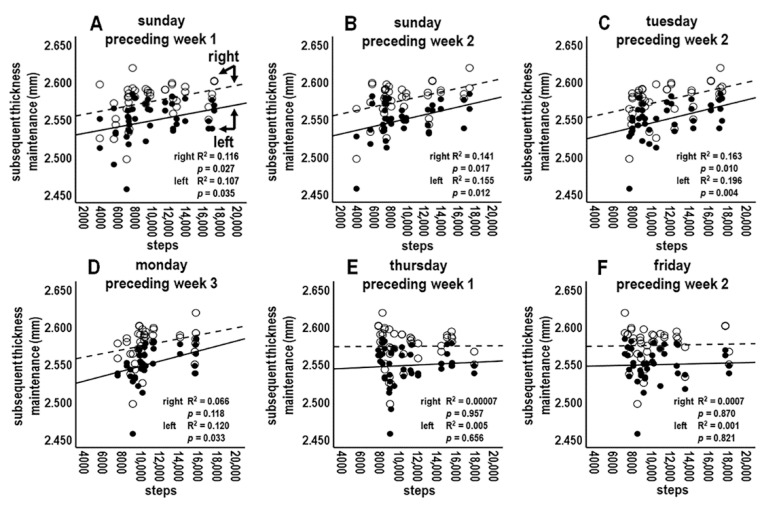
Scatterplots, linear regression lines, and associated R^2^ for relationships between steps during early week segment individual days of preceding weeks 1 (**A**), 2 (**B**,**C**), and 3 (**D**) and latter week segment individual days of preceding weeks 1 (**E**) and 2 (**F**) vs. subsequent thickness maintenances of separate right and left cortices. Identification of right and left cortices as indicated in (**A**) also applies to (**B**–**F**).

Preceding steps during individual early week segment days could be positively related to subsequent thickness maintenance of each cortex. For example, preceding early week segment days at which significant or trend-level positive relations were seen in Analysis 4 had further trend-level positive relations for each cortex (e.g., Figure 6A–C). Steps during preceding other early week segment days where trends for positive relations were seen in Analysis 4 had trend-level positive relations with one but not both cortices (e.g., Figure 6D).

Contrasting with the above positive trend relationships for preceding early week segment days, no relationships were seen between daily activity for preceding latter week segment days and thickness maintenances of each cortex (e.g., Figure 6E,F). Relationships for early week segment days noticeably differed from relationships for latter week segment days (e.g., compare the positive scatterplot distributions and regression lines for each cortex in Figure 6A–C to the flat distributions and regression lines for each cortex in Figure 6E,F).

#### 3.4.6. Question 1 Summary

The above analyses suggest preceding activity during (a) the prior 3 but not earlier weeks had a significant positive relationship with subsequent thickness maintenance (Figure 2C). In addition, preceding activity during (b) individual week periods of prior weeks 2–3 (Figure 3D), (c) early but not latter segments of prior weeks 1–3 (Figure 4F), and (d) early but not latter week individual days of weeks 1–3 (Figure 5I) had graded significant and/or trend-level positive relationships with subsequent thickness maintenance. Preceding activity relationships with subsequent thickness maintenance involved associations that were delayed/prolonged over shortest to longer times of respectively 4–7 days to 2–3 weeks (Figure 4F and Figure 5I). Trend-level positive relationships were seen for each cortex (Figure 6).

### 3.5. Question 2: Was Preceding Maintenance of Cortical Thickness Related to Subsequent Physical Activity and, if So, over What Times?

Question 2 examined physical activity and cortical thickness maintenance relationships in the reverse direction to that addressed in Question 1, using the approach of assessing broader to narrower periods of activity.

#### 3.5.1. Analysis 6: Preceding Thickness Maintenance vs. Activity for Subsequent Three-Week Periods (*n* = 4 Tests; Bonferroni Adjusted *p* ≤ 0.0125)

**Figure 7:**
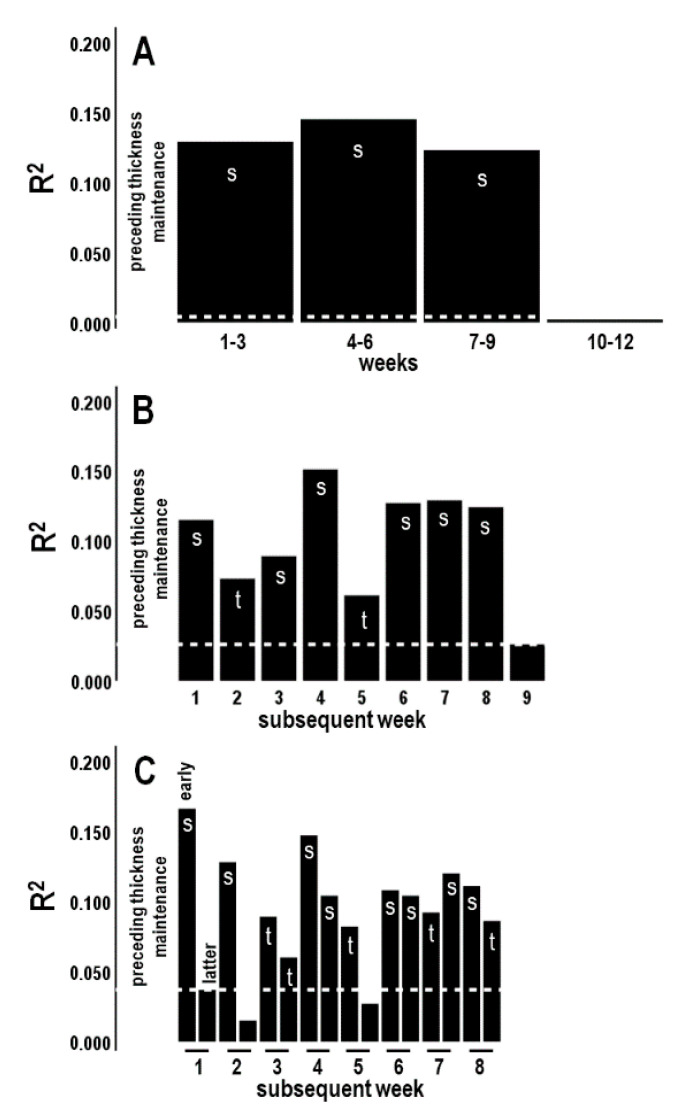
R^2^ profile summaries for preceding thickness maintenance vs. average steps/day during subsequent 3-week periods (**A**), individual week periods (**B**), and early- and latter-week segments (**C**). Significant (s) and trend (t)-level associations are indicated; measures at and below the dashed lines were not significant. In (**C**), sequence of early (left bar) and latter (right bar) week segments indicated for week 1 also applies to each other week.

Preceding thickness maintenance was significantly positively related to average steps/day over subsequent sequential 1st–3rd, 4th–6th, and 7th–9th three-week periods (Figure 7A). In contrast, preceding thickness maintenance was not related to activity over the subsequent 10th–12th three-week period (Figure 7A).

#### 3.5.2. Analysis 7: Preceding Thickness Maintenance vs. Activity for Subsequent Individual Weeks (*n* = 9; Bonferroni Adjusted *p* ≤ 0.005)

Preceding thickness maintenance was significantly positively related to average steps/day during the subsequent individual 1st, 3rd, 4th, and 6th–8th weeks and positively related at trend levels to activity during the subsequent 2nd and 5th weeks (Figure 7B; Table 2). In contrast, thickness maintenance was not related to activity during the 9th week (Figure 7B; Table 2). This suggested that preceding thickness maintenance had graded, delayed/prolonged relationships with activity during the subsequent first 8 individual-week periods.

#### 3.5.3. Analysis 8: Preceding Thickness Maintenance vs. Activity for Subsequent Week Segments (*n* = 16; Bonferroni Adjusted *p* ≤ 0.003) 

The above results led to tests of relationships between preceding thickness maintenance and activities during early week, i.e., Monday–Thursday, and latter week, i.e., Friday–Sunday, segments of the subsequent 8 weeks.

Preceding thickness maintenance was positively related to subsequent average steps/day for 81% (13/16) of the 16 total early and latter week segments, with 50% (8/16) significantly related, and 31% (5/16) related at trend levels (Table 3; Figure 7C). Relationships for the remaining 19% (3/16) were not significant (Table 3; Figure 7C).

The interval for initial positive relationships was short, and gradients in positive relationships were seen for early vs. latter week segment activities. Specifically, beginning with a significant positive relationship with activity during the early week segment of subsequent week 1, i.e., within the 1st–4th days after thickness maintenance measures, activity during early week segments of all other weeks also had significant or trend-level positive relationships with preceding thickness maintenance (100%, 8/8) (Table 3; Figure 7C). In addition, activity during a majority (62.5%, 5/8) of latter week segments had significant or trend-level positive relationships with preceding thickness maintenance (Table 3; Figure 7C).

These results suggest preceding thickness maintenance relationships with subsequent activity involved graded positive associations that were temporally delayed/prolonged, with shortest to longer times between associations of respectively 1–4 days to 8 weeks (Figure 7C).

Analyses of activity during separate days were not pursued based on thinking that individual day associations across 8 weeks would not provide more meaningful bracketing of relationships than these week segment results.

#### 3.5.4. Analysis 9: Left and Right Cortex Laterality Analyses (*n* = 6; Bonferroni Adjusted *p* ≤ 0.008)

The above analyses used pooled thickness maintenances of both hemispheres. To test if preceding thickness maintenances of each cortex could be related to subsequent activity, thickness maintenances of left and right cortices were considered separately. Because relationships for 3-week periods (Analysis 6) encompassed relationships for shorter periods (Analyses 7–8), activity for 3-week periods were assessed.

**Figure 8:**
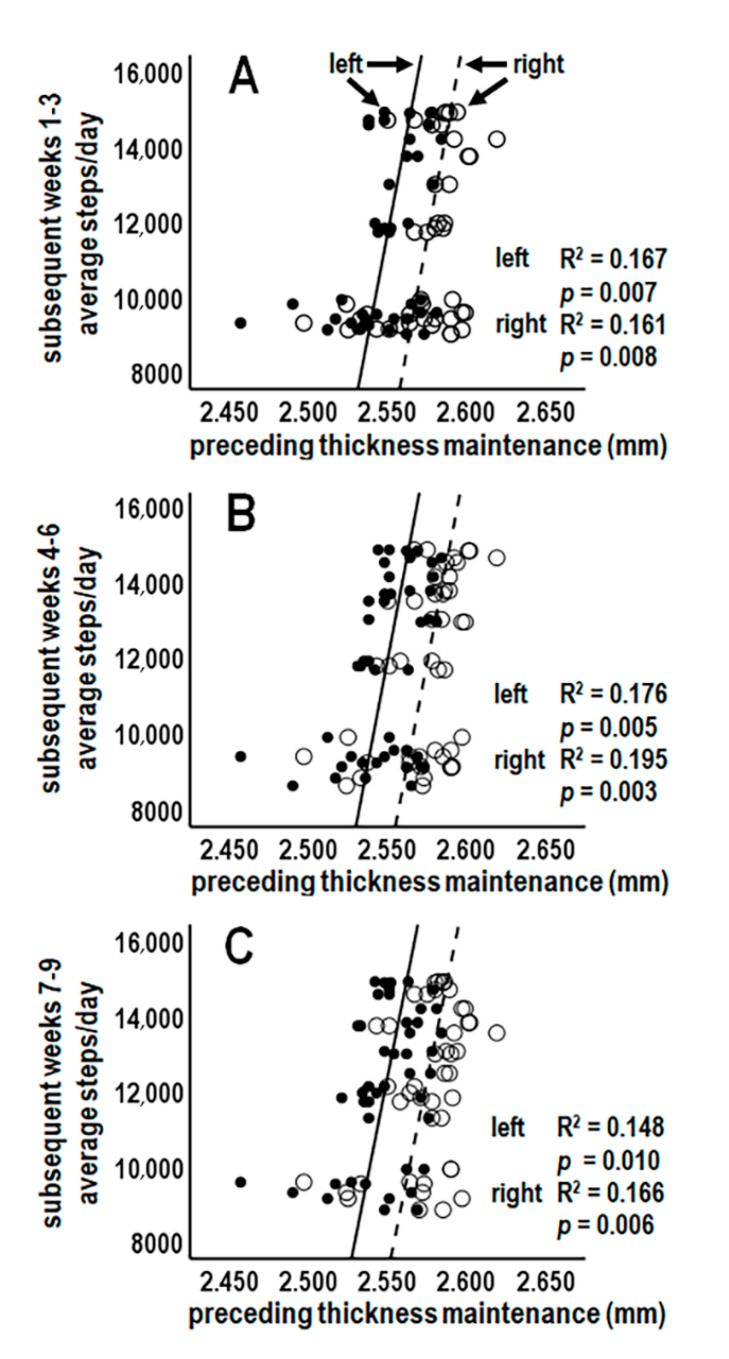
Scatterplots, linear regression lines, and associated R^2^ of relationships between preceding thickness maintenances of separate left and right cortices vs. average steps/day during subsequent weeks 1–3 (**A**), 4–6 (**B**), and 7–9 (**C**). The convention distinguishing right and left cortices in (**A**) also applies to (**B**,**C**).

Preceding thickness maintenances of the left and right cortices were each significantly positively related to subsequent mean activity over weeks 1–3 (Figure 8A) and 4–6 (Figure 8B). Over weeks 7–9, preceding thickness maintenance of the right cortex was significantly positively related to subsequent mean activity, whereas maintenance of the left cortex was positively related to activity at a trend level (Figure 8C). These results suggest preceding thickness maintenances of each cortex were positively related to subsequent activity.

#### 3.5.5. Question 2 Summary 

The above analyses suggest preceding thickness maintenance had significant positive relationships with activity during the subsequent (a) 1st–3rd, 4th–6th, and 7th–9th but not later week periods (Figure 7A) and graded significant to trend-level positive relationships with activity during (b) the related first 8 individual-week periods (Figure 7B) and (c) all early week segments and most latter week segments of these 8 weeks (Figure 7C). Preceding thickness maintenance to subsequent activity associations were graded and delayed/prolonged over shortest to longer times of respectively 1–4 days to 8 weeks (Figure 7C). Significant or trend-level positive relationships were seen for each cortex (Figure 8).

## 4. Discussion

### 4.1. Present Results

Maintenance of the adult cerebral cortex involves continuous structural rebuilding over day/week periods that is needed to revitalize structure. Normal maintenance of cortical thickness in an individual person potentially interacts with their physical activity but, to our knowledge, temporal interactions have not been studied at an individual person level. This study used micro-longitudinal tracking to address two questions regarding potential temporal interactions at an individual level. Given the absence of available data and frank uncertainty about whether or when interactions might occur in an individual, testing of activity from empirically varied times was necessary to address our questions.

Question 1: Was preceding physical activity related to subsequent maintenance of cortical thickness and, if so, over what times? In the studied individual, preceding physical activity over the prior 3 but not earlier weeks was significantly positively related to subsequent thickness maintenance. Graded significant and/or trend-level positive relationships were seen between preceding activity and subsequent thickness maintenance for activity that occurred during early but not latter segments of preceding weeks 1–3 and early but not latter week individual days of these weeks. Relationships in this direction involved associations that were delayed/prolonged over shortest to longer times of respectively 4–7 days to 2–3 weeks and that were bilateral, i.e., involved thickness maintenances of both cortices.

Question 2: Was preceding maintenance of cortical thickness related to subsequent physical activity and, if so, over what times? In the studied individual, preceding thickness maintenance was significantly positively related to physical activity that occurred during the subsequent 1st–3rd, 4th–6th, and 7th–9th but not latter week periods. Graded significant and/or trend-level positive relationships were seen between preceding thickness maintenance and activity for all individual-week periods of subsequent weeks 1–8 and for all early week segments and most latter week segments of these weeks. Relationships in this direction involved associations that were delayed/prolonged over shortest to longer times of respectively 1–4 days to 8 weeks and that were bilateral, i.e., involved thickness maintenances of both cortices.

In addressing Questions 1 and 2, we note that conservative Bonferroni adjustments were applied to reduce false positive results and that results that reached significant and trend levels of significance are considered and clearly distinguished. This is useful for defining associations where periods with conservatively defined significant relationships may have extended to yet earlier/later periods with trend-level relationships which, together, may comprise graded, delayed/prolonged associations that are meaningful from a micro-longitudinal, individual person perspective. Bonferroni-adjusted significance levels and distinction of periods with related significant and trend-level associations arguably permitted transparent consideration of this possibility without either exaggerating or overlooking associations.

### 4.2. Concepts from the Present Results

Taken together, the findings of Questions 1 and 2 suggest that the following concepts characterized temporal interactions between physical activity and cortical thickness maintenance in the studied individual.

*Interactions were bidirectional.* Preceding activity interacted with subsequent thickness maintenance and, conversely, preceding thickness maintenance interacted with subsequent activity. *Interactions were positive.* Interactions in both directions were consistently positive. *Interactions involved both cortices.* Each cortex was involved in interactions in both directions. *Interactions had limits.* Significant and trend-level relationships in the two directions ranged within modest *R^2^* magnitudes. This suggests that in each direction, the preceding factor did not, by itself, exclusively dictate the subsequent measure. *Interactions involved multi-week periods.* Interactions extended over periods of 3 (Question 1) and 8 (Question 2) weeks. *Interactions were graded.* There were gradients in interactions for different multi-week, week, week segment, and day periods as reflected by variability in scatterplot distributions, related regression lines, and significant or trend-level relationships. *Interactions were temporally asymmetric.* Interactions in the preceding thickness maintenance to subsequent activity direction had a shorter initial onset (1–4 days) and longer duration (8 weeks) than the initial onset (4–7 days) and duration (3 weeks) of interactions in the reverse preceding activity to subsequent thickness maintenance direction. *Interactions were prolonged and/or delayed.* The findings of significant relationships across weeks and that periods with significant or trend-level relationships were separated by intervening periods that had no relationships suggest interactions were delayed and/or prolonged. *Interactions were continuous.* Interactions extended micro-longitudinally across the study. Overall, the observed bidirectional positive interactions suggest that physical activity may have continuous protective effects on cortical substrate maintenance which, in turn, promotes physical activity.

The above concepts come from one person, which raises the generalizability issue. Due to individual person specificity (see Implication 2 below), selection of a generally representative individual would appear to be very difficult. However, it is unlikely the present study serendipitously assessed the only individual to whom one or more of these concepts pertain; thus, they arguably generalize to some extent to other individuals. On further consideration, physical activity and cortical thickness vary across individuals, thus indicating that different expressions of the above concepts or different concepts apply in other individuals. This suggests the merit of exploring generalizability with further micro-longitudinal individual person analyses.

### 4.3. Comparison of the Present Individual-Focused Concepts to Existing Group-Based Concepts of Interactions between Cortical Thickness and Physical Activity 

Cortical thickness has rarely been studied with a micro-longitudinal individual person analysis design, i.e., using intraindividual analysis of sequential thickness measures taken from an individual person at regular short intervals over a period of several months [8,9]. Recent reviews point to a need for brain studies using such designs [10,11,12]. Moreover, this type of design has not been used to investigate interactions between cortical thickness and physical activity.

Existing studies have, however, extensively assessed relationships between cortical thickness and physical activity with group-focused analyses. Concepts of relationships from the present individual-focused findings in some ways are similar to and predicted, but in other ways have not been detected, by group-focused work.

Similarities in concepts from group- and the present individual-focused findings include the following.

*Positive relationships*. Most group studies report positive relationships between physical activity and cortical thickness [4,5,6,26,27,28,29,30,31], with fewer studies finding either mixed positive and negative [32] or no [33,34] relationships. Aligning with many group-based results, interactions in the studied individual were positive.

*Relationship bilaterality.* Group-based work has reported relationships between physical activity and cortical thicknesses of both cortices [27,28,30,32]. Similarly, interactions were bilateral in the presently studied individual. 

*Long periods for relationships.* Group analyses indicate activity over long periods can be related to cortical thickness [4,5,26,28,29,30,31,32]. Consistent with these findings, in the studied individual, interactions extended over multi-week periods.

*Relationship limitations.* Group work suggests physical activity operates collectively with other factors to affect cortical thickness [5,27]. In the studied individual, the strengths of interactions had limitations that did not reflect exclusive control relationships and, thus, likely involved influences of other factors (see below *Implication 1*).

Beyond the above similarities in group- vs. the present individual-based concepts, group-based work has paid little or no attention to other concepts of interactions that were resolved with the present micro-longitudinal individual-focused analyses, including the following. 

*Interaction directionality.* Establishing direction(s) of interaction(s) between cortical structure and physical activity is an important focus of health neuroscience [35,36,37,38]. Group-based studies indicate that physical activity affects cortical thickness [4,5,6,27,28,29,30,31,32]. To our knowledge, only one group study has examined the reverse direction (“reverse causality”) possibility that cortical thickness affects physical activity [39]. The novel findings of that study suggest cortical thickness affects subsequent adherence to physical activity. Given limited attention to reverse direction effects, group-based conceptualization of directionality of physical activity and cortical thickness relationships is still being worked out. Distinction of direction(s) of interaction(s) is arguably improved with micro-longitudinal tracking and corresponding within-individual tests for independent relationships in each direction over sequentially continuous time periods. The present study provides such tests and suggests interactions in the studied individual were bidirectional.

*Temporal interaction dynamics.* Of further importance, group-based work has not attempted to address concepts that pertain to micro-longitudinal temporal characteristics regarding the presently observed *(a) gradients, (b) time asymmetries, (c) delays and/or prolongations,* and *(d)* ongoing *continuous* nature of interactions. The present findings of interactions that are: continuously ongoing, graded in strength over prolonged/delayed day to multi-week periods, and asymmetric in the two directions provide original insight into temporal interaction dynamics that can operate at an individual person level.

In summary, concepts from the present study agree with concepts from group analyses in some respects. In addition, the present findings complement current understanding by identifying concepts of temporal interactions that have received little or no attention in group work but that are arguably relevant for understanding relationships between maintenance of cortical thickness and physical activity at an individual level. This raises the possibility that an individual-focused approach can contribute to individualized precision medicine tailoring of physical activity recommendations by applying individual-focused concepts that have not been recognized or readily resolved by group-based work.

### 4.4. Normality of Interactions

This issue requires considering whether the individual’s steps/day activity and thickness maintenance measures were or were not normal.

*The studied individual’s steps/day activity.* What constitutes normal daily steps for a particular individual person remains enigmatic and must be indirectly judged from diverse findings from population/group-focused work, including the following.

*First*, adults normally take ≈3000–18,000+ steps/day, with group average rates in different countries ranging, e.g., from ≈5000–9600 steps/day [40,41,42]. The world-wide population’s average rate is estimated to be ≈4961 steps/day [43].

*Second*, classification schemes for group data categorize steps/day activity. For example, in one scheme, “sedentary” and “low activity” categories ranged from <5000–7499 steps/day, a “moderate activity” category ranged from 7500–9999, and “active” and “very active” categories ranged from ≥10,000 and ≥12,500 steps/day [44].

*Third*, longstanding mass media and national promotions suggest a population goal for adults to walk 10,000 steps/day [45,46,47,48,49].

*Fourth*, numerous reports suggest that average rates in the range of ≈4400–10,000 steps/day are associated with increased longevity and health benefits [50,51,52,53,54,55,56,57,58,59,60,61]. Less studied, activity above this range also has beneficial health effects [54,59,61,62,63,64].

*Finally*, it is proposed that a population-level threshold of ≈7000–8000 steps/day relates to public health guidelines for getting 30 min/day or 150 min/week of moderate-to-vigorous physical activity [40,57,65,66]. There also appears to be variation in this relationship across individuals [67].

The above work, although comprehensive with respect to normal group activity and public health guidelines, was not designed to identify what constitutes a normal number of steps for any specific person. However, the findings from this work currently provide the most relevant indices for gauging if an individual’s daily step activity is within normal expectations. The studied individual averaged 11,159 (±3354) steps/day; his pattern of activity over days, weeks, and months reflected a continuously active profile (Figure 1C); and his measures equaled or exceeded the above normal population/group indices. This suggests his steps/day activity fit normal expectations.

*The studied individual’s cortical thickness maintenance measures.* As previously reported [8,13] and briefly reviewed in Methods, the studied individual’s medical history and daily health measures attested to his good health and fitness over life and during the study. Moreover, we have previously presented detailed comparisons which show that his hemispheric thickness mean and variation measures were encompassed within the range of hemispheric thickness mean and variation measures reported in 11 studies that applied FreeSurfer thickness measurement procedures in normal adult groups of his age and younger [13]. From this, his cortical thickness maintenance measures arguably fit normal expectations.

*Summary.* The above views suggest that the studied individual’s steps/day activity and cortical thickness measures were within normal expectations and that the observed results likely reflect normal interactions.

### 4.5. Implications 

The present findings have useful implications.

Implication 1: *At an individual person level, normal maintenance of cortical thickness, physical activity, and their micro-longitudinal temporal interactions are likely affected by multiple factors.*

The observed R^2^ magnitudes suggest that maintenance of cortical thickness in the studied individual was not exclusively dictated by preceding physical activity and that physical activity was not exclusively dictated by preceding maintenance of thickness.

Consistent with these possibilities, group-level work has shown cortical thickness to be associated with, e.g., body mass index [68], genetic [69], hormonal [70], stress [71], diet [72], allostatic load [73], time of day [74], gut microbiome [75], cardiovascular [76], and metabolic [77] factors.

Similarly, group-level work indicates that physical activity has associations with multiple factors, including, e.g., diet [78], gut microbiome [79], emotion regulation [80], fitness [81], weather [82], and metabolic [83] factors. Adding to group-level findings, studies using micro-longitudinal tracking and within-individual analyses have shown physical activity’s associations with, e.g., sleep [84,85,86,87] and stress [88].

Moreover, directly pertinent to the present findings, cortical thickness maintenance in the studied individual has been shown to be associated with both sleep duration and thickness maintenance during prior days/weeks [9,13]. Thus, other factors were likely in play in the studied individual and arguably co-influenced and perhaps limited the presently observed interactions. Taken with other recent proposals [89], this suggests a need for precision medicine research on how maintenance of cortical thickness, physical activity, and other factors micro-longitudinally co-interact at an individual level and whether, e.g., cortical thickness maintenance may mediate/moderate or be mediated/moderated by co-interactions.

Implication 2: *Identifying concepts of micro-longitudinal temporal interactions between cortical thickness maintenance and lifestyle factors like physical activity at the individual level can improve individualized precision medicine tailoring of cortical structural maintenance and neurocognitive health.*

To our knowledge, no previous work has investigated micro-longitudinal temporal interactions between fluctuations in cortical thickness maintenance and physical activity at an individual person level. This is an overlooked area of investigation.

Precision medicine has interests in maximizing cortical structural and related neurocognitive health, in part through individualized tailoring of potentially influential lifestyle factors like physical activity that may continuously affect brain maintenance. This focus on the individual person derives from a recognition of the importance of human variability and individual specificity.

Pertinent to the present investigation and precision medicine interests, cortical structure, including thickness, varies across individuals [90,91,92,93] and is individual specific [94,95,96,97,98,99]. Analogous to cortical thickness, physical activity also varies across individuals [100,101,102,103,104] and is individual specific [84,105,106,107].

At a group level, physical activity has been shown to be associated with cortical thickness [6,7,27], and activity interventions can change cortical thickness [4,5,31,108,109].

Differences in cortical structure, including thickness, are associated with differences in neurocognitive functions [92,94,110,111,112,113]. Similarly, although contrary results have been reported [114,115,116], much work indicates physical activity also has associations with neurocognitive functions [117,118,119,120,121,122,123,124,125,126].

The present findings complement the above group-level work by providing a beginning perspective on intraindividual micro-longitudinal temporal interactions between physical activity and cortical thickness maintenance that potentially contribute to individual specificity of physical activity, cortical thickness, and neurocognition. Recognition of these interactions may be useful for personalized tailoring of physical activity. It is possible, for example, that knowledge of an individual’s temporal delays/prolongations in interaction between preceding physical activity and subsequent thickness maintenance and between preceding thickness maintenance and subsequent activity might be used to inform tailoring of personalized recommendations for activity scheduling that will optimally affect that individual’s (a) thickness maintenance, (b) neurocognitive health, and (c) self-motivation to maintain healthful physical activity. Considered with recent views on personalized health maintenance [1,3,38], there is hope that further understanding of these interactions can improve individualized precision medicine tailoring of activity, brain, and neurocognitive health.

## 5. Limitations

This study has clear limitations. (1) The data and related interaction concepts are from one person. (2) Cortical structure is assessed only in terms of hemispheric mean thickness. (3) Physical activity is assessed only in terms of steps/day. (4) The data pertain to free-living and not intervention-related conditions. (5) The findings suggest “if-then”, i.e., if this occurs, then this follows, and not “cause-effect” interactions. (6) This work is an exploratory starting, not finishing, point for understanding interactions between maintenance of cortical structure and physical activity at an individual person level.

## 6. Conclusions

Maintenance of cerebral cortical structure, reflected in part by cortical thickness, is necessary to maintain neurocognitive health. Precision medicine has interests in using personalized adjustment of lifestyle factors, including physical activity, to optimize maintenance of a person’s cortical thickness and related neurocognitive health. However, whether or how maintenance of cortical thickness in an individual person micro-longitudinally interacts with their physical activity remains unclear. The present study used an unconventional micro-longitudinal tracking approach to assess temporal interactions between cortical thickness maintenance and physical activity over empirically varied time windows in a healthy adult man. These novel person-focused findings in some ways are predicted, but in other ways remain undetected, by existing group-focused work. We suggest that an understanding of person-focused interactions can complement group-focused findings and improve individualized precision medicine tailoring of cortical structural maintenance, physical activity, and related neurocognitive health.

## Figures and Tables

**Table 1:** Relationships between steps each day of preceding weeks 1, 2, and 3 vs. subsequent thickness maintenance.

	Sun	Mon	Tue	Wed	Thu	Fri	Sat
Preceding week 1							
R^2^	0.088	0.030	0.007	0.055	0.001	0.033	0.005
r	0.296	0.172	0.084	0.234	0.035	0.180	0.072
*p*	0.006	0.117	0.449	0.032	0.749	0.100	0.515
	T	NS	NS	T	NS	NS	NS
Preceding week 2							
R^2^	0.114	0.037	0.138	0.064	0.009	0.0007	0.012
r	0.337	0.192	0.371	0.254	0.097	0.028	0.111
*p*	0.002	0.088	0.001	0.023	0.390	0.806	0.316
	S	NS	S	T	NS	NS	NS
Preceding week 3							
R^2^	0.059	0.070	0.039	0.062	0.0008	0.039	0.006
r	0.244	0.265	0.197	0.249	0.029	0.197	0.077
*p*	0.034	0.021	0.088	0.030	0.801	0.089	0.499
	T	T	NS	T	NS	NS	NS

R^2^ = linear regression coefficient; r = Pearson correlation; *p* = significance; S = significant; T = trend; NS = not significant.

**Table 2:** Relationships between preceding thickness maintenance and average daily steps for each of subsequent weeks 1–9.

	1st Week	2nd Week	3rd Week	4th Week	5th Week	6th Week	7th Week	8th Week	9th Week
R^2^	0.115	0.073	0.089	0.151	0.061	0.127	0.129	0.124	0.026
r	0.339	0.270	0.298	0.389	0.246	0.357	0.359	0.352	0.161
*p*	0.002	0.011	0.005	0.001	0.021	0.001	0.001	0.001	0.135
	S	T	S	S	T	S	S	S	NS

R^2^ = linear regression coefficient; r = Pearson correlation; *p* = significance; S = significant; T = trend; NS = not significant.

**Table 3:** Relationships between preceding thickness maintenance and average daily steps for early and latter week segments of subsequent weeks 1–8.

	1st Week		2nd Week		3rd Week		4th Week		5th Week		6th Week		7th Week		8th Week	
	Early	Latter	Early	Latter	Early	Latter	Early	Latter	Early	Latter	Early	Latter	Early	Latter	Early	Latter
R^2^	0.166	0.037	0.128	0.015	0.089	0.060	0.147	0.104	0.082	0.027	0.108	0.104	0.092	0.120	0.111	0.086
r	0.407	0.192	0.358	0.122	0.298	0.245	0.383	0.322	0.286	0.166	0.329	0.323	0.304	0.347	0.333	0.293
*p*	0.001	0.080	0.001	0.256	0.005	0.021	0.001	0.002	0.007	0.123	0.002	0.002	0.004	0.001	0.002	0.006
	S	NS	S	NS	T	T	S	S	T	NS	S	S	T	S	S	T

Early: early week (Monday–Thursday) segment; Latter: latter week (Friday–Sunday) segment; R^2^ = linear regression coefficient; r = Pearson correlation; *p* = significance; S = significant; T = trend; NS = not significant.

## Data Availability

Raw data are not publicly available due to subject privacy and ethics concerns. Address questions to J.W. at john.wall@utoledo.edu.
